# Supplementary material for: Water Consumption during a School Day and Children’s Short-Term Cognitive Performance: The CogniDROP Randomized Intervention Trial
Source: Nutrients. 2020 May 2;12(5):1297. doi: 10.3390/nu12051297 (PMC7282257; doi:10.3390/nu12051297)
Supplement: Supplementary file 1 [file nutrients-12-01297-s001.pdf]

Supplement

Table 1. Results of the cognitive tasks by study arm.

	Intervention			Control			
	x̃	25th	75th	x̃	25th	75th	*p* Value
**Switch Task**		***n* = 120**			***n* = 105**		
Switch costs (s)	29.5	19.4	44.6	26.4	14.6	46.1	0.276
Visual search letters (s)	36.4	31.2	45.2	34.0	30.0	41.2	0.085
Visual search numbers (s)	53.1	46.1	61.2	51.9	45.6	61.1	0.700
Visual search switch (s)	93.0	76.0	115.9	86.4	70.3	103.8	0.109
**Corsi Block**		***n* = 136**			***n* = 114**		
Longest Path (n)	5.0	4.0	6.0	5.0	4.3	6.0	0.734
Correct immediate block span (n)	6.0	4.0	8.0	6.0	4.0	8.0	0.671
Score	10.5	7.0	16.3	11.5	7.0	15.0	0.849
**2-back**		***n* = 135**			***n* = 113**		
Ratio of missings (%)	33.3	19.0	42.9	33.3	23.8	47.6	0.491
Ratio of false alarms (%)	11.2	7.1	25.9	12.9	7.1	23.5	0.328
RT (ms)[a]	466	403	550	465	382	551	0.601
Count of correct events (n)	88.5	76.0	94.0	86.0	77.0	92.0	0.349
**Flanker Task**		***n* = 107**			***n* = 82**		
RT slowing (ms)	79.7	56.5	98.8	72.6	51.9	100.0	0.532
Difference error rate (%)	17.1	8.6	28.6	17.1	10.7	28.6	0.648
Count of false alarms (n)	8.0	3.0	15.0	7.0	3.0	19.0	0.378

Mann–Whitney *U*-test, [a]*t*-test, data are presented as x̃ = Median, 25th and 75th percentiles, level of significance was set to $p \leq 0.05$.

Table 2. Results of the switch and flanker task including all participants.

	Study Arm[a]		Categorie[b]	
	Intervention (*n* = 136)	Control (*n* = 114)	Water in mL	% of TWI
**Switch Task**				
Switch costs	0.613		0.176	0.618
Visual search letters	0.534		0.311	0.908
Visual search numbers	0.896		0.590	0.924
Visual search switch	0.200		0.540	0.522
**Flanker Task**				
RT slowing (ms)	0.403		0.909	0.802
Difference error rate	0.223		0.573	0.494
Count of false alarms	0.720		0.134	0.155

[a]Mann-Whitney U test; [b]Kruskal-Wallis test; data are presented as a *p* value; $p \leq 0.05$; TWI = total water intake.

Table 3. Relationship between urine color, thirst and cognitive performance.

		Thirst[a]	
	Ucol[b]	yes	no
**Switch task**	***n* = 223**	***n* = 81**	***n* = 142**
Switch costs (s)[b]	0.917	0.205	
Visual search letters (s)[c]	0.583	0.029	
Visual search numbers (s)	0.909	0.171	
Visual search switch (s)	0.822	0.017	
**Corsi block-tapping task**	***n* = 248**	***n* = 88**	***n* = 160**
Longest Path (n)	0.816	0.647	
Correct immediate block span (n)	0.956	0.480	
Score	0.966	0.409	
**2-back task**	***n* = 246**	***n* = 87**	***n* = 159**
Ratio of missings (%)	0.530	0.437	
Ratio of false alarms (%)	0.413	0.154	
RT (ms)	0.884	0.173	
Count of correct events (n)	0.323	0.225	
**Flanker task**	***n* = 189**	***n* = 73**	***n* = 115**
RT slowing (ms)	0.069	0.817	
Difference error rate (%)	0.685	0.738	
Count of false alarms (n)	0.925	0.853	

[a]Mann–Whitney U-test, [b] Spearman's correlation, level of significance was set to $p \leq 0.05$.
